# 4161 Contraceptive Decision Making Among Women with Diabetes Mellitus: A Mixed Methods Study

**DOI:** 10.1017/cts.2020.378

**Published:** 2020-07-29

**Authors:** Emily Hazel Johnson, Justine Wu

**Affiliations:** 1 University of Michigan School of Medicine; 2 University of Michigan

## Abstract

OBJECTIVES/GOALS: Women with diabetes (DM) have higher rates of unplanned pregnancy and pregnancy complications, indicating a need for DM-specific counseling. Our aim is to use the Health Belief Model (HBM), widely applied to DM interventions, to create a conceptual model of contraceptive decision-making for women with DM. METHODS/STUDY POPULATION: Our convergent mixed methods study integrates quantitative survey data and qualitative interview data from women aged 18-50 with DM. We will interview until theoretical saturation is achieved. Descriptive statistics will be calculated to summarize demographics, health history, and contraceptive use and knowledge. Using a grounded theory approach, qualitative analysis will be conducted by JW and EJ with special focus on exploring HBM constructs. A joint display will be used to integrate key quantitative variables by qualitative themes. Finally, we will aim to implement these HBM constructs within the context of contraceptive decision-making for women with DM. RESULTS/ANTICIPATED RESULTS: To date, we have interviewed 16 women with average age of 35 and an equal mix of type 1 vs. type 2 DM and those who identify their health as poor/fair vs. good/very good. The following HBM domains have emerged as relevant to contraceptive decision-making: perceived threats, perceived barriers, cues to action and self-efficacy. An analysis of the cues to action domain suggests that patients’ responses to provider counseling are substantially dependent on provider tone and awareness of patient context. Effective cues were delivered supportively with focus on health promotion, while ineffective cues focused on adverse outcomes or were delivered to women not at risk of pregnancy. DISCUSSION/SIGNIFICANCE OF IMPACT: The application of the HBM to contraceptive decision making among women with DM will provide insight into critical factors that inform both patient and provider attitudes and behaviors and can be targeted for future interventions to reduce unplanned pregnancy in this vulnerable population.

